# Contributions to the Pathological Anatomy of Acute Delirium

**Published:** 1879-08

**Authors:** 


Contributions to the Pathological Anatomy of Acute Delirium.—Jehn (*Arch. Psych.* viii., page 594) has had the opportunity of observing and studying four cases. The first patient was ill for twenty-two days, and eight days before he died gangrene of the right leg set in, beginning at the foot and spreading rapidly over the whole limb. The right forearm, from the hand to the elbow upwards, was also similarly affected. At the necropsy an unexpected complication was met with in the shape of a hard tumor, of the size of a nut, on the left side of the pons, which seemed to spring from the acoustic nerve; the ganglia of the sympathetic cardiac plexus were partly degenerated, and the cortex of both kidneys showed fatty degeneration.

The second case lasted for sixteen days. A few days before death phlegmonous inflammation of the right foot set in, and on the night preceding the end, the patient's back, abdomen and legs were covered with numerous pustules. At the necropsy the latter were found to communicate with abscesses under the skin. The liver was partly in a state of fatty degeneration, and the capsules of the kidneys very adherent, the cortex being of a yellowish tinge.

In the third case the patient was delirious for twenty-six days. In the course of the last six days a gangrenous phlegmon of the right leg set in. The liver was swollen and in a partial condition of fatty degeneration, the cortical layer of the kidneys of a yellowish hue and adhering to the capsules. The author considers this case, as well as the fourth, as being closely allied to acute paralysis.

In the latter, acute delirium set in towards the end of an illness which had lasted four months. It broke out while the patient was under mercurial treatment for syphilis, and lasted for fourteen days. Here also a gangrenous inflammation was observed similar to those which have been described above, which broke out in the vicinity of an open syphilitic ulcer on the right thigh. At the *post mortem* examination the posterior columns were found in a state of grey degeneration.

In comparing the results of the microscopical examination in all four cases, they were found to be alike in several points. The pia mater was always thick, dark, and of the consistence and appearance of jelly; the vessels, especially in the grey matter, were all more or less in a state of fatty degeneration, and traces of small hemorrhages could be detected in their vicinity.

The nervous system seemed to have been affected secondarily, the affection manifesting itself in a fatty degeneration of the cells of the ganglia. In some cases the former had entirely vanished, and in their place only a large mass of fatty globules could be seen, while in other cells the change had hitherto confined itself to the nucleus, increasing it in size. The author is of opinion that such cases ought to be considered as acute meningo-encephalitis.—*London Medical Journal.*

